# TRAIL gene 1595C/T polymorphisms contribute to the susceptibility and severity of intervertebral disc degeneration: a data synthesis

**DOI:** 10.1186/s12891-017-1916-3

**Published:** 2017-12-29

**Authors:** Qi-ling Yuan, Liang Liu, Yong-song Cai, Yin-gang Zhang

**Affiliations:** Department of Orthopaedics of the First Affiliated Hospital, Medical School, Xi’an Jiaotong University, No. 261 of West Yanta Road, Xi’an, Shaanxi 710061 China

**Keywords:** TRAIL gene, Polymorphisms, Intervertebral disc degeneration, Meta-analysis

## Abstract

**Background:**

Studies have investigated the correlation between tumor necrosis factor related apoptosis-inducing ligand (TRAIL) gene polymorphisms and the susceptibility and severity of intervertebral disc degeneration (IDD), but the results were inconsistent. To evaluate the specific relationship, we performed a meta-analysis to clarify the controversies.

**Methods:**

Four databases were searched, and the pooled results were presented as odds ratios (ORs) with 95% confidence intervals (CIs).

**Results:**

Three case-control studies from Han Chinese were included (565 cases and 427 controls). All the included studies reported TRAIL 1595C/T gene polymorphisms. The recessive model (CC vs. CT + TT) was the optimal model, which demonstrated a significant relationship between 1595C/T polymorphisms and increased IDD risk (OR = 2.18, 1.45 to 3.27, *P* = 0.000). No significant heterogeneity was found in the recessive model (I^2^ = 48.6%, *P* = 0.143). Patients with lower grade IDD had more genotypes or alleles including 1595TT genotype (grade II vs. grade III: OR = 2.12, 1.18 to 3.83, *P* = 0.012; grade III vs. grade IV: OR = 2.59, 1.29 to 5.22, *P* = 0.007) and 1595 T allele (grade II vs. grade III: OR = 1.91, 1.43 to 2.55, *P* = 0.000; grade II vs. grade IV: OR = 2.46, 0.94 to 1.76, *P* = 0.000).

**Conclusions:**

There is a significant relationship between 1595C/T polymorphisms and the susceptibility and severity of IDD in Han Chinese. Patients with lower grade IDD had higher frequency of the 1595TT genotype and 1595 T allele.

**Electronic supplementary material:**

The online version of this article (doi: 10.1186/s12891-017-1916-3) contains supplementary material, which is available to authorized users.

## Background

Lower back pain is a very common condition. According to the report, 84% of people in the general population suffer from lower back pain in some way, and many people are permanently disabled due to this condition [1]. Intervertebral disc degeneration (IDD) is commonly thought to be an important cause of back pain. Intervertebral discs are soft tissue structures that can absorb and distribute applied loads between vertebral bodies and provide flexibility for the spine [2, 3]. In the past 200 years, lower back pain has been associated with IDD, aging, excessive physical labor and inflammatory cytokines, and more recently with genetic factors. Intervertebral cell loss caused by apoptosis plays an important role in the pathogenesis of IDD [4].

Tumor necrosis factor and apoptotic induction ligands (TRAILs) are transmembrane proteins that belong to tumor necrosis factor ligands. In transformed cells and cancer cells, TRAIL preferentially did apoptosis inductions by TRAIL-R2/DR5 and TRAIL-R1/DR4 receptors [5]. Three single nucleotide polymorphisms (SNPs) have been found in the nucleotides of the 3′ untranslated region of TRAIL gene, 1595, 1588 and 1525, respectively [6]. The expression of TRAIL gene in human disc is correlated with IDD, suggesting that the TRAIL gene may play a role in IDD mechanism [7]. Hence, this study further discussed the relationship between TRAIL gene variation and IDD pathogenesis, which provides a basis for clinical treatment.

Although a series of studies [8–10] have examined the link between the common TRAIL gene SNPs and the risk of IDD, the overall results are uncertain. Given these inconsistent results, we need to summarize the current data on the correlation between TRAIL gene polymorphism and IDD risk. Therefore, we conducted a meta-analysis of all eligible studies to assess the correlation between TRAIL gene polymorphism and IDD’s susceptibility and severity.

## Methods

### Search strategy

We conducted a computer-based retrieval using the following English and Chinese databases: PubMed, Embase, the China National Knowledge Infrastructure database, and the China Biology Medical Literature database. We did also try to identify additional records through other sources, such as the reference lists in the relevant research reports. The search was conducted from the inception of each database on May 1st, 2016. Our search strategies (Additional file 1: Table S1) were iteratively developed using the following words: tumor necrosis factor related apoptosis-inducing ligand, TRAIL, polymorphism*, SNP*, and disc degeneration.

### Study selection

The following types of studies were included in the meta-analysis: (1) the study design was case-control study; (2) the populations of interests were human subjects; (3) the outcome was evaluation of the correlation between TRAIL polymorphisms and IDD risk; (4) sufficient data such that the odds ratios (ORs) and 95% confidence intervals (CIs) could be calculated; (5) and no publication language was restricted.

Accordingly, the exclusion criteria were defined as follows: (1) data overlapping with previous publications; (2) comments, reviews, or animal studies; or (3) studies with genotype frequencies that were not detailed. Eligible studies were reviewed independently by two reviewers (QLY and LL) according to the inclusion criteria. Disagreements were resolved by discussion.

### Data extraction

Two reviewers (QLY and YSC) independently extracted the data from the eligible studies. The data were collected as follows: (1) name of the first author; (2) publication year; (3) country where the study was conducted; (4) ethnicity of the enrolled subjects; (5) gender and age of study participants; (6) numbers of cases and controls; (7) method of genotyping; and (8) genotype frequency in cases and controls. The two reviewers reached a consensus on all the data.

### Risk of bias assessment

We extracted and modified the methodological quality assessment scale based on a previous study [11]. On this scale (Additional file 1: Table S2), eight items were carefully checked: representativeness of cases, representativeness of controls, ascertainment of IDD, ascertainment of control, genotyping examination, Hardy-Weinberg equilibrium (HWE), association assessment, and response rate. Total scores ranged from 0 (worst) to 15 (best). The quality of the included studies was independently checked by two reviewers (WTW and FS). In the case of any disagreements, a consensus was achieved after discussion.

### Analysis

The chi-square test was applied to detect the *P* value of HWE in the control group, and if the *P* value was greater than 0.05, which satisfied the HWE. The correlation strength between TRAIL polymorphism and IDD risk was calculated by means of odds ratio (OR) and 95% confidence interval (CI). We merged ORs from every trial to reckon up the pooled ORs. Z-test was used to judge if the pooled ORs were statistically significant, and the *P* value lower than 0.05 indicated a significance. Different genetic models were used.

We used Q statistic to detect the statistical heterogeneity, and a *P* value smaller than 0.10 was regarded as significance; similarly, I^2^ > 50% also indicated a significance [12]. For a more conservative estimation, we used a random effects model (Mantel-Haenszel method) to combine our data. Jack-knife analyses as sensitivity analyses were used to any single study which influenced the observed heterogeneity disproportionately. Both Begg’s test [13] and Egger’ regression test [14] were resorted to detect possible pubication bias, and a sinificance was recongnized while *P* < 0.05.

Shapiro-Wilk W test was applied to estimate whether the models were suitable for the data in our meta-analysis [15]. The metagen order in STATA was used to identify the optimal genetic model [16]. Considering that a wrong genetic model could result in vast of power loss, we chose the best model before calculating the OR for each model, which could avoid an increased type I error rate derived from testing all possible models [17].

We estimated the disc degeneration severity of IDD patients with different TRAIL genotypes and alleles, and the variance-stabilizing double arcsine transformation [18] was used to pool prevalence.

STATA 14 (Stata, CollegeStation, TX) was used to do all these analyses above. All *P*-values were two-sided.

## Results

### Characteristics of studies

**Fig. 1:**
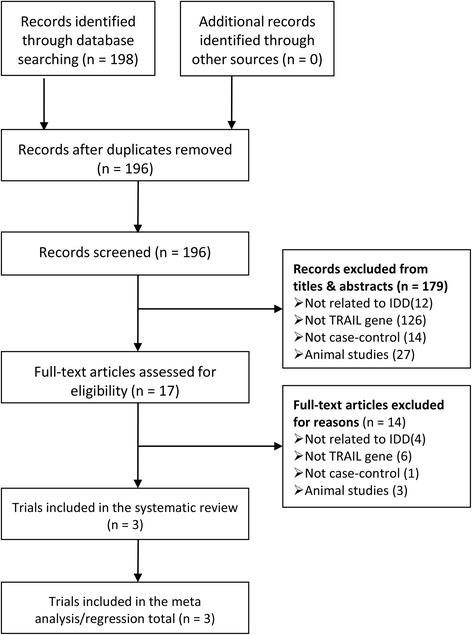
Flow chart

Our search strategy identified 198 potentially eligible records (Fig. 1). A total of 2 duplicates were excluded, and 179 additional records were also excluded based on the titles or abstracts for reasons such as the presence of a disorder that was not related to IDD, no TRAIL genes were evaluated, or studies that used non-human subjects. After full-text articles were assessed for eligibility, 14 records were excluded for not meeting our inclusion criteria. Finally, three case-control studies [8–10] with 565 cases and 427 controls published in English meeting our inclusion criteria were included in our meta-analysis. All the studies had been conducted in China, and all the enrolled subjects were Han Chinese. All the included studies reported TRAIL 1595C/T gene polymorphisms. Polymerase chain reaction–restriction fragment length polymorphism (PCR-RFLP) was applied in all included studies. Genomic DNA was isolated from blood samples in all included studies. Magnetic resonance imaging was used to diagnose IDD in all the cases, and Schneiderman’s classification was used to rate the severity or grade of IDD. In all three studies, the controls were in HWE. The quality scores of the included trials ranged from 10 to 11, which indicated a good quality (Additional file 1: Table S3). The detailed characteristics of each study are shown in Tables 1 and 2.

**Table 1:** Characteristics of individual studies

Authors	Year	Country	Ethnicity	Gender (M/F)		Age, mean (SD)		Matching	Control source	Genotyping method	IDD diagnosis	IDD severity standard	Study quality
				Case	Control	Case	Control						
Zhang [10]	2015	China	Han Chinese	198/114	121/75	49.54(8.87)	48.65(8.12)	Age and gender	Healthy subjects	PCR-RFLP	Symptoms, MRI	Schneiderman’s classification	11
Du [8]	2015	China	Han Chinese	73/80	62/69	40.2(5.1)	38.6(5.0)	Age and gender	Healthy subjects	PCR-RFLP	Symptoms, MRI	Schneiderman’s classification	11
Xu [9]	2015	China	Han Chinese	54/46	58/42	59	49	Age and gender	Healthy subjects	PCR- RFLP	Symptoms, MRI	Schneiderman’s classification	10

*M* male, *F* female, *SD* standard deviation, *IDD* intervertebral disc degeneration, *PCR-RFLP* polymerase chain reaction–restriction fragment length polymorphism, *MRI* magnetic resonance imaging

**Table 2:** Distribution of genotypes and alleles of TRAIL 1595C/T gene polymorphisms between cases and controls

Authors	Case					Control					P* values for HWE
	Genotype			Allele		Genotype			Allele		
	CC	CT	TT	C	T	CC	CT	TT	C	T	
Zhang [10]	192	79	41	463	161	70	88	38	228	164	0.2782
Du [8]	46	71	36	163	143	22	66	43	110	152	0.6954
Xu [9]	37	46	17	120	80	29	43	28	101	99	0.1618

*HWE* Hardy-Weinberg equilibrium

* *P* value for HWE in control group was calculated by χ^2^ test and *P* value >0.05 was considered as fulfilling HWE

### Optimal genetic model

The goodness-of-fit test was used to evaluate how the models fit the data in the meta-analysis, in which the Shapiro-Wilk W test for normal distribution was applied in STATA. We found that all *P* values were >0.05, indicating that these data were normally distributed and suitable for genetic models in the meta-analysis. And then, the metagen command in STATA was used to identify the optimal genetic model for TRAIL 1595C/T gene polymorphisms. This command was based on the ideology of logistic regression, in which every different genotype was considered as different independent variable. In this logistic regression model, OR_1_ (CT vs. TT) = exp. (θ_1_), OR_2_ (CC vs. TT) = exp. (θ_2_), and the null hypothesis (H_0_) was as follow: θ_1_ = θ_2_. As a result, we obtained a *P* value of <0.001 (<0.05), suggesting that the results support H_0_, indicating that there was a correlation between the TRAIL gene and IDD. Meanwhile, we also obtained a result as follows: θ_1_ = 0.073 (−0.096 to 0.243, *P* = 0.396 > 0.05) and θ_2_ = 0.382 (0.211 to 0.554, *P* = 0.000 < 0.05), so θ_1_ = 0 and θ_2_ > 0, indicating that the recessive model (CC vs. CT + TT) was the optimal model.

### Association between 1595C/T polymorphisms and IDD susceptibility

**Fig. 2:**
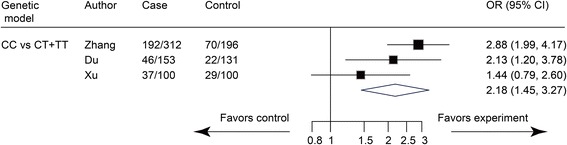
Meta-analysis of the association between 1595C/T polymorphisms and IDD susceptibility. CI, confidence interval; IDD, intervertebral disc degeneration; OR, odds ratio

The recessive model (CC vs. CT + TT) was the optimal model, which showed a significant relationship between 1595C/T polymorphisms and increased IDD risk (OR = 2.18, 95% CI: 1.45 to 3.27, *P* = 0.000) (Fig. 2). No significant heterogeneity among included studies was found in the recessive model (I^2^ = 48.6%, *P* = 0.143).

### Association between 1595C/T polymorphisms and IDD severity

**Fig. 3:**
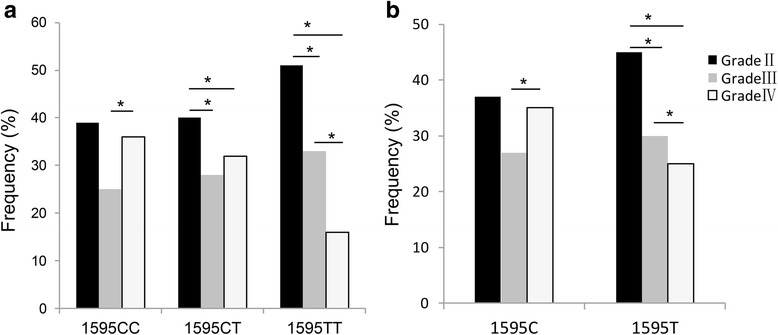
Association between 1595C/T polymorphisms and IDD severity. **a** The results revealed that patients with lower grade IDD had more genotypes including 1595TT genotype (grade II vs grade III, *P* = 0.012; grade III vs grade IV, *P* = 0.007); **b** The results revealed that patients with lower grade IDD had more alleles including 1595T allele (grade II vs grade II, *P* = 0.000; grade II vs grade IV, *P* = 0.000). IDD, intervertebral disc degeneration; * indicating a significant difference between groups (*P* value < 0.05)

To assess the severity of disc degeneration among IDD patients with different 1595C/T genotypes and alleles, we pooled prevalence estimates with the variance-stabilizing double arcsine transformation. Meta-analyses of disc degeneration grades among IDD patients with different 1595C/T genotypes and alleles are shown in Table 3 and Fig. 3. The results (Fig. 3) revealed that patients with lower grade IDD had more genotypes or alleles including 1595TT genotypes (grade II vs grade III: OR = 2.12, 1.18 to 3.83, *P* = 0.012; grade III vs grade IV: OR = 2.59, 1.29 to 5.22, *P* = 0.007) (Fig. 3a) and 1595 T allele (grade II vs grade III: OR = 1.91, 1.43 to 2.55, *P* = 0.000; grade II vs grade IV: OR = 2.46, 0.94 to 1.76, *P* = 0.000) (Fig. 3b).

**Table 3:** Meta-analysis of disc degeneration grades among IDD patients with different TRAIL 1595C/T genotypes and alleles

Author	Genotypes or alleles	n	Grade II	Grade III	Grade IV	χ^2^ or Z value ^a^	*P* value
Zhang [10]	CC	192	94 (48.96%)	42 (21.88%)	56 (29.17%)		
	CT	79	48 (60.76%)	22 (27.85%)	9 (11.39%)		
	TT	41	20 (48.78%)	15 (36.59%)	6 (14.63%)	175.400	<0.001
	C	463	236 (50.97%)	106 (22.89%)	121 (26.13%)		
	T	161	88 (54.66%)	52 (32.30%)	21 (13.04%)	−19.612	<0.001
Du [8]	CC	46	14 (30.43%)	12 (26.09%)	20 (43.48%)		
	CT	71	22 (30.99%)	18 (25.35%)	31 (43.66%)		
	TT	36	20 (55.56%)	9 (25.00%)	7 (19.44%)	66.501	<0.001
	C	163	50 (30.67%)	42 (25.77%)	71 (43.56%)		
	T	143	62 (43.36%)	36 (25.17%)	45 (31.47%)	−5.346	<0.001
Xu [9]	CC	37	11 (29.73%)	12 (32.43%)	14 (37.84%)		
	CT	46	13 (28.26%)	15 (32.61%)	18 (39.13%)		
	TT	17	8 47.06%)	7 (41.18%)	2 (11.76%)	69.084	<0.001
	C	101	30 (29.70%)	34 (33.66%)	37 (36.63%)		
	T	99	35 (35.35%)	33 (33.33%)	31 (31.31%)	−2.081	0.037
Meta-analyses ^b^	CC	275	108 (39.27%)	68 (24.73%)	99 (36.00%)		
	CT	196	79 (40.31%)	55 (28.06%)	62 3(31.63%)		
	TT	94	48 (51.06%)	31 (32.98%)	15 (15.96%)	395.200	<0.001
	C	730	273 (37.40%)	199 (27.26%)	258 (35.34%)		
	T	400	180 (45.00)	120 (30.00%)	100 (25.00%)	−28.366	<0.001

*TRAIL* tumor necrosis factor related apoptosis-inducing ligand, *IDD* intervertebral disc degeneration

^a^ χ^2^ value was calculated by Kruskal-Wallis H, and Z value by Mann-Whitney Test

^b^ Double-arcsine-transformed prevalence method was used to perform meta-analyses of prevalences

### Sensitivity analysis and publication bias

Sensitivity analysis confirmed the presence of an association between 1595C/T and IDD risk. We excluded one study at a time, and the omission of any single study resulted in the same significant difference as was detected in the overall meta-analysis (as shown in Table 4, all *P* values <0.05). In addition, all the point estimates of ORs (1.76, 2.12 and 2.64, respectively) were in the range of the 95% CI of the overall meta-analysis (1.45 to 3.27), suggesting that the results of this meta-analysis were robust.

**Table 4:** Sensitivity analysis with each study omitted (recessive model)

Study omitted	OR (95% CI)	*P* value
Zhang	1.76 (1.17, 2.66)	0.007
Du	2.12 (1.08, 4.17)	0.030
Xu	2.64 (1.93, 3.60)	0.000
Overall meta-analysis	2.18 (1.45, 3.27)	0.000

*OR* odds ratio, *CI* confidence interval

Publication bias in the recessive model was tested. No evidence of publication bias was observed (Egger’s test *P* = 0.276; Begg’s test *P* = 0.296).

## Discussion

### Major findings

Our meta-analysis revealed the differential distribution of genotype/allele frequencies of the TRAIL 1595C/T gene among different clinical grades of IDD patients in the Han Chinese population. The occurrence rate of 1595TT genotype and 1595 T allele was lower in advanced-stage patients (Fig. 3). These results indicated the possible value of TRAIL gene polymorphisms in predicting the risk and severity of IDD, although the underlying mechanisms of this relationship were unknown.

### Comparison with other studies

The mechanisms involved in IDD are still unclear, and many internal and external causes may contribute to histopathological changes in intervertebral disc [19]. IDD is known to be a multifactorial disease, and genetic factors play an important role in IDD [20]. Gene polymorphisms were shown to be related to IDD risk, included genes for interleukins [21], collagen [22] and apoptosis-associated genes. Recent studies revealed the association between different polymorphisms in apoptosis-associated genes with the risk of IDD. The Ex5 + 32 G/A polymorphism of the caspase 9 gene was reported to be related to IDD in the Han Chinese population [23]. In addition, Guo et al. reported that the 1263 GG genotype of the 1263A/G polymorphism in the caspase 9 gene was significantly correlated to an increased risk of developing discogenic low back pain [24]. Moreover, associations were also observed between other apoptosis-related gene polymorphisms (e.g., FAS and FAS ligand [25], BCL-2 [26]) and the susceptibility and severity of IDD in the Han Chinese population. Among the involved genes, the TRAIL gene has recently become a focus in IDD research.

TRAIL functions as a critical immune regulatory factor and is involved in immune homeostasis, immune surveillance and immune modulation [27]. Associations between TRAIL gene polymorphisms and ovarian cancer have been described in some researches. One study [28] reported significantly decreased TRAIL protein expression in malignant ovarian epithelial tumor cells compared to benign ovary tumor cells and normal tissues. Further analysis identified lower TRAIL protein expression in patients with stage III/IV ovarian cancer compared to patients with stage I/II ovarian cancer. TRAIL has also been shown to be involved in the pathogenesis of IDD. One study [7] identified that the expression of TRAIL and its receptors DR4 and DR5 was associated with the progression of IDD. Moreover, degenerated discs presented a much higher percentage of TRAIL- and DR5-positive cells, as well as increased staining intensity, compared with normal tissue [29]. These findings indicated the important role of the TRAIL gene in the mechanism of IDD, which were consistent with our findings. However, further studies should be conducted to explore the exact mechanism of TRAIL in the development of IDD.

### Strengths and weaknesses

A main strength of this study is that this is the first meta-analysis to address the correlation between TRAIL gene polymorphisms and the susceptibility and severity of IDD. Our systematic review was conducted in strict accordance with the PRISMA statement [30]. A series of rigorous statistical approaches were applied to obtain reliable results according to the Cochrane Handbook 5.1.0 [31], which included the Shapiro-Wilk W test for normal distribution, identification of the optimal genetic model, and variance-stabilizing double arcsine transformation for pooling prevalence estimates of the severity of IDD.

Although there are some strengths for our study, some limitations require consideration. The main weaknesses of this study are that few studies were eligible and that the sample sizes were relatively small. The small number of studies meant that the statistical power to detect differences was suboptimal. However, the pooled results in our review were more reliable than the results in each of the individual studies. Specifically, all the included trials only reported data on 1595 C/T, thus we were unable to examine the association of other SNPs (e.g., 1588) of the TRAIL gene with IDD risk. Moreover, the demographic data and the IDD disease-related factors were not reported in detail and so few trials were included, which limited further analyses (e.g., subgroup analysis, meta-regression analysis). In addition, although no evidence of publication bias was evident according to Egger’s test and Begg’s test, the possibility of publication bias might still exist because there were only three studies and only studies published in English and Chinese were retrieved. In addition, all the included studies were conducted in China, and all the subjects were Han Chinese, thus the external validity of our study is limited. Finally, IDD is a multifactorial disease, and the association between TRAIL gene polymorphisms and IDD risk or severity might be magnified or diminished by gene-gene, gene-protein and/or gene-environment interactions. However, this issue could not be addressed herein because of the limited availability of individual patient data, such as family history, performance of manual labor and other environmental factors. Overall, these limitations could lead to false-positive findings, thus, additional prospective studies with larger sample sizes including participants of other ethnic populations are warranted.

## Conclusion

Our findings suggest that there is a significant relationship between 1595C/T polymorphisms and the susceptibility and severity of IDD in the Han Chinese. Patients with lower grade IDD exhibited higher frequency of 1595TT genotypes and 1595 T alleles. These findings should be validated in prospective studies with a larger population including patients of other ethnicities.

## Additional file

Additional file 1: Supplementary Tables and Figures. (DOCX 303 kb)

## Abbreviations

CI: Confidence interval
HWE: Hardy-Weinberg equilibrium
IDD: Intervertebral disc degeneration
OR: Odds ratio
SNP: Single nucleotide polymorphism
TRAIL: Tumor necrosis factor related apoptosis-inducing ligand
