# Supplementary material for: TRAIL gene 1595C/T polymorphisms contribute to the susceptibility and severity of intervertebral disc degeneration: a data synthesis
Source: BMC Musculoskelet Disord. 2017 Dec 29;18:555. doi: 10.1186/s12891-017-1916-3 (PMC5747187; doi:10.1186/s12891-017-1916-3)
Supplement: Additional file 1: — Supplementary Tables and Figures. (DOCX 303 kb) [file 12891_2017_1916_MOESM1_ESM.docx]

TRAIL gene 1595C/T polymorphisms contribute to the susceptibility and severity of intervertebral disc degeneration: a data synthesis

**Authors:**

Qi-ling Yuan^1^, Liang Liu^1^, Yong-song Cai^1^ & Yin-gang Zhang^1*^

**Affiliations:**

^1^Department of Orthopaedics of the First Affiliated Hospital, Medical School, Xi’an Jiaotong University, No. 261 of West Yanta Road, Xi’an 710061, Shaanxi, China

***Corresponding Author:**

Yin-gang Zhang; Mailing address: Department of Orthopaedics of the First Affiliated Hospital, Medical School, Xi’an Jiaotong University, No. 261 of West Yanta Road, Xi’an 710061, China; E-mail: [zyingang@mail.xjtu.edu.cn](mailto:zyingang@mail.xjtu.edu.cn); Tel.: +86 029 85323935

**Table S1** Search strategy in PUBMED

| Item | Key words |
| --- | --- |
| #1 | (((tumor necrosis factor related apoptosis-inducing ligand[Title/Abstract]) OR TRAIL[Title/Abstract]) OR ((polymorphism[Title/Abstract]) OR polymorphisms[Title/Abstract])) OR ((((SNP[Title/Abstract]) OR SNPs[Title/Abstract]) OR single-nucleotide polymorphism[Title/Abstract]) OR single-nucleotide polymorphisms[Title/Abstract]) |
| #2 | (((((((intervertebral disc degeneration[MeSH Terms]) OR disc degeneration[Title/Abstract]) OR disk degeneration[Title/Abstract]) OR inervertebral disk degeneration[MeSH Terms])) OR ((degenerative[Title/Abstract]) AND ((disc[Title/Abstract]) OR disk[Title/Abstract])))) OR ((((((((((((((((((((low back pain[Title/Abstract]) OR lower back pain[Title/Abstract]) OR lumbago[Title/Abstract]) OR discogenic back pain[Title/Abstract]) OR discogenic pain[Title/Abstract]) OR lumbodorsal[Title/Abstract]) OR neck pain[Title/Abstract]) OR cervical pain[Title/Abstract]) OR cervicodynia[Title/Abstract]) OR disc herniation[Title/Abstract]) OR disk herniation[Title/Abstract]) OR disc prolapse[Title/Abstract]) OR disc protrusion[Title/Abstract]) OR dorsalgia[Title/Abstract]) OR radicular pain[Title/Abstract]) OR root pain[Title/Abstract]) OR nerve root pain[Title/Abstract]) OR cervical spondylosis[Title/Abstract]) OR cervical spondylopathy[Title/Abstract]) OR cervical sydrome[Title/Abstract]) |
| #3 | #1 AND #2 |

**Table S2** Methodological quality assessment scale

| Item | Criteria | Score |
| --- | --- | --- |
| Q1 | Representativeness of cases |  |
|  | Consecutive/randomly selected from case population with clearly defined sampling frame | 2 |
|  | Consecutive/randomly selected from case population without clearly defined sampling frame or with extensive inclusion/exclusion criteria | 1 |
|  | No method of selection described | 0 |
| Q2 | Representativeness of controls |  |
|  | Controls were consecutive/randomly drawn from the same sampling frame (ward/community) as cases | 2 |
|  | Controls were consecutive/randomly drawn from a different sampling frame as cases | 1 |
|  | Not described | 0 |
| Q3 | Ascertainment of Intervertebral disc degeneration (IDD) |  |
|  | Clearly described objective criteria for diagnosis of IDD | 2 |
|  | Diagnosis of IDD by patient self-report or by patient history | 1 |
|  | Not described | 0 |
| Q4 | Ascertainment of control |  |
|  | Controls were tested to screen out IDD, i.e., MRI | 2 |
|  | Controls were subjects who did not report IDD; no objective testing | 1 |
|  | Not described | 0 |
| Q5 | Genotyping examination |  |
|  | Genotyping done under ‘‘blinded’’ condition | 1 |
|  | Unblinded or not mentioned | 0 |
| Q6 | Hardy-Weinberg equilibrium (HWE) |  |
|  | HWE in control group | 2 |
|  | Hardy-Weinberg disequilibrium in control group | 1 |
|  | No checking for HWE | 0 |
| Q7 | Association assessment |  |
|  | Assess association between genotypes and IDD with appropriate statistics and adjustment for confounders | 2 |
|  | Assess association between genotypes and IDD with appropriate statistics without adjustment for confounders | 1 |
|  | Inappropriate statistics used | 0 |
| Q8 | Response rate |  |
|  | Response rates for both groups are the same, i.e., to within 5% | 2 |
|  | Response rates are different, between 5% and 10% | 1 |
|  | Response rates are more than 10% different, or no mention of response rates | 0 |
| TOTAL |  |  |

Q, question.

**Table S3** Result of methodological quality of included studies

| Study | Q1 | Q2 | Q3 | Q4 | Q5 | Q6 | Q7 | Q8 | Total |
| --- | --- | --- | --- | --- | --- | --- | --- | --- | --- |
| Zhang | 2 | 2 | 2 | 2 | 0 | 2 | 1 | 0 | 11 |
| Du | 2 | 2 | 2 | 2 | 0 | 2 | 1 | 0 | 11 |
| Xu | 1 | 2 | 2 | 2 | 0 | 2 | 1 | 0 | 10 |

Q, question.


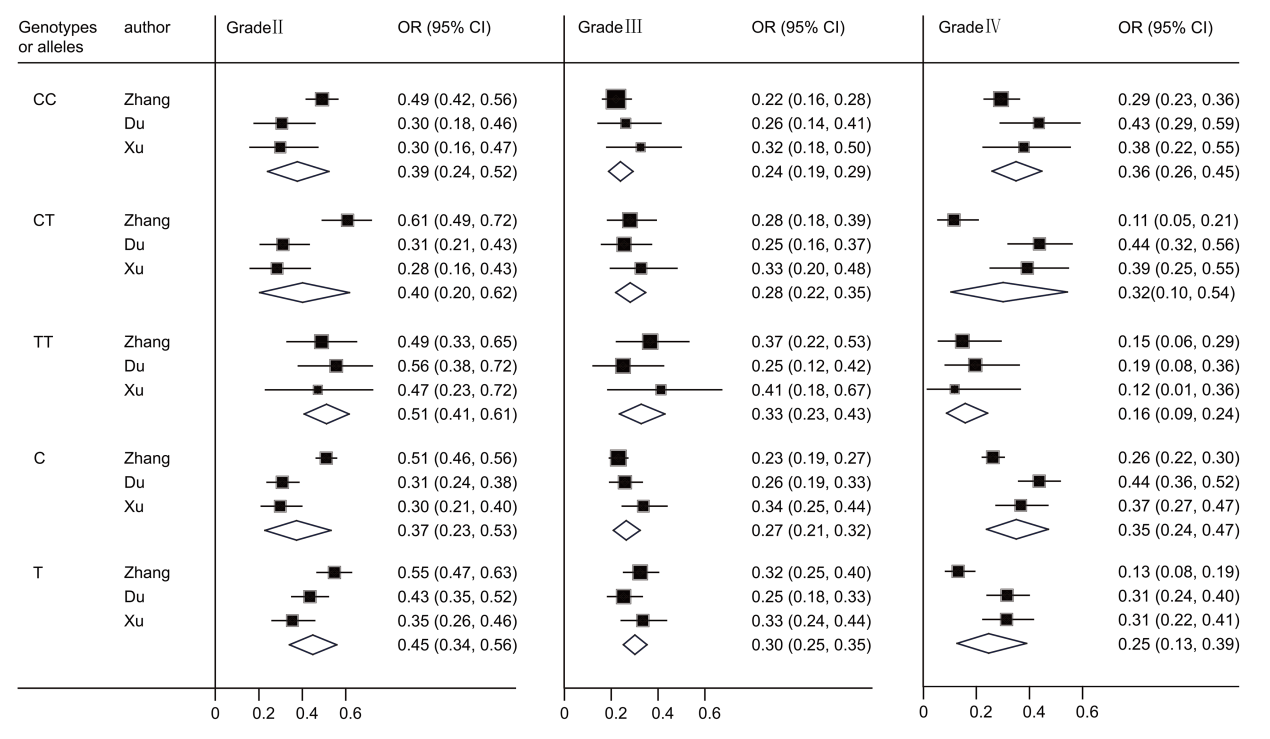


**Figure S1** Meta-analysis of the association between 1595C/T polymorphisms and IDD severity. CI, confidence interval; IDD, intervertebral disc degeneration; OR, odds ratio.

Text S1 - Checklist of items to include when reporting a systematic review or meta-analysis

| Section/topic | # | Checklist item | Reported on page # |
| --- | --- | --- | --- |
| **TITLE** | | | |
| Title | 1 | Identify the report as a systematic review, meta-analysis, or both. | 1 |
| **ABSTRACT** | | | |
| Structured summary | 2 | Provide a structured summary including, as applicable: background; objectives; data sources; study eligibility criteria, participants, and interventions; study appraisal and synthesis methods; results; limitations; conclusions and implications of key findings; systematic review registration number. | 2 |
| **INTRODUCTION** | | | |
| Rationale | 3 | Describe the rationale for the review in the context of what is already known. | 3 |
| Objectives | 4 | Provide an explicit statement of questions being addressed with reference to participants, interventions, comparisons, outcomes, and study design (PICOS). | 3 |
| **METHODS** | | | |
| Protocol and registration | 5 | Indicate if a review protocol exists, if and where it can be accessed (e.g., Web address), and, if available, provide registration information including registration number. |  |
| Eligibility criteria | 6 | Specify study characteristics (e.g., PICOS, length of follow-up) and report characteristics (e.g., years considered, language, publication status) used as criteria for eligibility, giving rationale. | 4 |
| Information sources | 7 | Describe all information sources (e.g., databases with dates of coverage, contact with study authors to identify additional studies) in the search and date last searched. | 4 |
| Search | 8 | Present full electronic search strategy for at least one database, including any limits used, such that it could be repeated. | 4 and supplementary file Table S1 |
| Study selection | 9 | State the process for selecting studies (i.e., screening, eligibility, included in systematic review, and, if applicable, included in the meta-analysis). | 4 |
| Data collection process | 10 | Describe method of data extraction from reports (e.g., piloted forms, independently, in duplicate) and any processes for obtaining and confirming data from investigators. | 4-5 |
| Data items | 11 | List and define all variables for which data were sought (e.g., PICOS, funding sources) and any assumptions and simplifications made. | 4-5 |
| Risk of bias in individual studies | 12 | Describe methods used for assessing risk of bias of individual studies (including specification of whether this was done at the study or outcome level), and how this information is to be used in any data synthesis. | 5 |
| Summary measures | 13 | State the principal summary measures (e.g., risk ratio, difference in means). | 5 |
| Synthesis of results | 14 | Describe the methods of handling data and combining results of studies, if done, including measures of consistency (e.g., I^2^) for each meta-analysis. | 5-6 |
| Risk of bias across studies | 15 | Specify any assessment of risk of bias that may affect the cumulative evidence (e.g., publication bias, selective reporting within studies). | 5 |
| Additional analyses | 16 | Describe methods of additional analyses (e.g., sensitivity or subgroup analyses, meta-regression), if done, indicating which were pre-specified. | 6 |
| RESULTS | | | |
| Study selection | 17 | Give numbers of studies screened, assessed for eligibility, and included in the review, with reasons for exclusions at each stage, ideally with a flow diagram. | 6-7 |
| Study characteristics | 18 | For each study, present characteristics for which data were extracted (e.g., study size, PICOS, follow-up period) and provide the citations. | 6-7 |
| Risk of bias within studies | 19 | Present data on risk of bias of each study and, if available, any outcome-level assessment (see Item 12). | 7 and supplementary file Table S3 |
| Results of individual studies | 20 | For all outcomes considered (benefits or harms), present, for each study: (a) simple summary data for each intervention group and (b) effect estimates and confidence intervals, ideally with a forest plot. | 7 and Table 1, 2, 3 and Fig. 2, 3, 4 |
| Synthesis of results | 21 | Present results of each meta-analysis done, including confidence intervals and measures of consistency. | 7-8 |
| Risk of bias across studies | 22 | Present results of any assessment of risk of bias across studies (see Item 15). | Table 1 |
| Additional analysis | 23 | Give results of additional analyses, if done (e.g., sensitivity or subgroup analyses, meta-regression [see Item 16]). | 8-9 |
| DISCUSSION | | | |
| Summary of evidence | 24 | Summarize the main findings including the strength of evidence for each main outcome; consider their relevance to key groups (e.g., health care providers, users, and policy makers). | 9 |
| Limitations | 25 | Discuss limitations at study and outcome level (e.g., risk of bias), and at review level (e.g., incomplete retrieval of identified research, reporting bias). | 11 |
| Conclusions | 26 | Provide a general interpretation of the results in the context of other evidence, and implications for future research. | 11-12 |
| FUNDING | | | |
| Funding | 27 | Describe sources of funding for the systematic review and other support (e.g., supply of data); role of funders for the systematic review. | 13 |
